# GAH-TNet: A Graph Attention-Based Hierarchical Temporal Network for EEG Motor Imagery Decoding

**DOI:** 10.3390/brainsci15080883

**Published:** 2025-08-19

**Authors:** Qiulei Han, Yan Sun, Hongbiao Ye, Ze Song, Jian Zhao, Lijuan Shi, Zhejun Kuang

**Affiliations:** 1 College of Computer Science and Technology, Changchun University, Changchun 130022, China; sunyan.ee@outlook.com (Y.S.); ye_hb1109@163.com (H.Y.); szfsy520@gmail.com (Z.S.); zhaojian@ccu.edu.cn (J.Z.); shilj@ccu.edu.cn (L.S.); kuangzhejun@ccu.edu.cn (Z.K.); 2 Key Laboratory of Intelligent Rehabilitation and Barrier-Free for the Disabled (Ministry of Education), Changchun University, Changchun 130022, China; 3 Jilin Provincial Key Laboratory of Human Health Status Identification & Function Enhancement, Changchun 130022, China; 4 Jilin Rehabilitation Equipment and Technology Engineering Research Center for the Disabled, Changchun 130022, China

**Keywords:** brain–computer interface, motor imagery, deep learning, graph neural network, attention mechanism

## Abstract

Background: Brain–computer interfaces (BCIs) based on motor imagery (MI) offer promising solutions for motor rehabilitation and communication. However, electroencephalography (EEG) signals are often characterized by low signal-to-noise ratios, strong non-stationarity, and significant inter-subject variability, which pose significant challenges for accurate decoding. Existing methods often struggle to simultaneously model the spatial interactions between EEG channels, the local fine-grained features within signals, and global semantic patterns. Methods: To address this, we propose the graph attention-based hierarchical temporal network (GAH-TNet), which integrates spatial graph attention modeling with hierarchical temporal feature encoding. Specifically, we design the graph attention temporal encoding block (GATE). The graph attention mechanism is used to model spatial dependencies between EEG channels and encode short-term temporal dynamic features. Subsequently, a hierarchical attention-guided deep temporal feature encoding block (HADTE) is introduced, which extracts local fine-grained and global long-term dependency features through two-stage attention and temporal convolution. Finally, a fully connected classifier is used to obtain the classification results. The proposed model is evaluated on two publicly available MI-EEG datasets. Results: Our method outperforms multiple existing state-of-the-art methods in classification accuracy. On the BCI IV 2a dataset, the average classification accuracy reaches 86.84%, and on BCI IV 2b, it reaches 89.15%. Ablation experiments validate the complementary roles of GATE and HADTE in modeling. Additionally, the model exhibits good generalization ability across subjects. Conclusions: This framework effectively captures the spatio-temporal dynamic characteristics and topological structure of MI-EEG signals. This hierarchical and interpretable framework provides a new approach for improving decoding performance in EEG motor imagery tasks.

## 1. Introduction

Motor imagery-based brain–computer interfaces (MI-BCIs) offer a novel solution for motor rehabilitation and communication [1,2,3]. Research indicates that MI-BCIs have been widely applied to restore motor function in patients with neuromuscular injuries, such as upper limb rehabilitation following stroke or spinal cord injury [4,5]. In addition, they have been widely used to control assistive devices such as wheelchairs, prosthetics, and speech synthesis systems, providing patients with new means of interaction [6,7].

With their high spatiotemporal resolution and non-invasive nature, MI-BCIs have become a research focus in neural rehabilitation and assistive communication [8,9,10]. However, electroencephalography (EEG) signals have low signal-to-noise ratios, are non-stationary, and are significantly influenced by individual differences among participants [11]. Therefore, the stable extraction of movement-related features from complex and noisy EEG recordings remains a major challenge in MI-BCI research [12,13].

Traditional machine learning methods have been extensively widely applied in MI-BCI, typically involving handcrafted feature extraction followed by classification with a linear classifier. Among these, the common space pattern (CSP) [14,15,16] is the most commonly used spatial feature extraction method, which enhances the differences between the two types of MI signals by identifying electrode-weighted vectors. In the classification stage, methods such as linear discriminant analysis (LDA) [17,18] and support vector machines (SVMs) [19,20] are commonly employed. However, these methods rely on manual feature design and have inherent limitations. For example, CSP is highly sensitive to bandwidth and the number of channels, leading to significant performance degradation under channel-constrained or noisy conditions; linear classifiers like LDA require manual feature extraction, making it difficult to automatically capture complex spatiotemporal features. Consequently, traditional methods have limitations in extracting spatiotemporal collaborative features from MI-EEG, which restricts both classification accuracy and robustness.

To overcome the limitations of traditional methods, researchers have recently introduced various deep learning architectures to automatically learn high-level feature representations of MI-EEG [21,22]. Convolutional neural networks (CNNs) have been widely applied in this field. For example, compact CNNs such as Shallow ConvNet [23] and EEGNet [24] can directly extract temporal features from raw EEG data. CNN-based models are effective at capturing local spatial patterns; however, their limited receptive fields restrict their ability to model long-range temporal dependencies. Therefore, some methods employ temporal convolutional networks (TCNs) [25] to expand the receptive field and linearly capture longer temporal dependencies. Representative examples include TCNet-Fusion [26] and EEG-TCNet [27], which extend the TCN architecture and achieve notable improvements in feature extraction capability and decoding accuracy.

On the other hand, self-attention mechanisms have been increasingly incorporated into MI-EEG decoding due to their powerful capabilities to capture long-range dependencies and global contextual information [28,29]. For instance, EEG-Conformer combines convolutional modules with multi-head self-attention mechanisms, enabling simultaneous extraction of local temporal features and global dependency information, thereby enhancing the model’s ability to capture complex EEG dynamics [30]. The attention-guided temporal convolutional network (ATCNet) further enhances adaptability to non-stationary EEG signals by regulating the feature extraction process through temporal attention, leading to improved decoding performance and robustness [31]. Moreover, EEG-TransNet integrates CNN and Transformer modules, enhancing the perception of global semantic information through multi-scale temporal modeling, and demonstrates superior decoding accuracy with strong cross-subject generalization [32].

Although the above methods have made significant progress, the spatio-temporal characteristics of MI-EEG remain insufficiently captured. Traditional methods cannot automatically learn rich nonlinear dynamics, and pure CNN or TCN models struggle to simultaneously model local and global spatio-temporal dependencies. Furthermore, most methods neglect the influence of electrode spatial topology on signal correlations, whereas graph neural networks can effectively model non-Euclidean topological relationships between electrodes, enhancing the representation of spatial dependencies [33]. For example, the multi-view GCN model MGCANet specifically constructs a graph convolutional structure based on brain region topology and introduces self-attention to expand the temporal receptive field, enabling joint modeling of cross-channel spatial dependencies and temporal features [34]. Similarly, the three-layer graph attention network (GAT) utilizes phase-locked value (PLV) to construct EEG structure, enhances cross-channel dynamic connection modeling capabilities through multi-layer attention aggregation mechanisms, and improves the ability to express nonlinear features [35]. Research indicates that the integration of graph structure modeling and hierarchical temporal feature extraction holds significant application potential and theoretical value in MI-EEG decoding.

**Figure 1:**
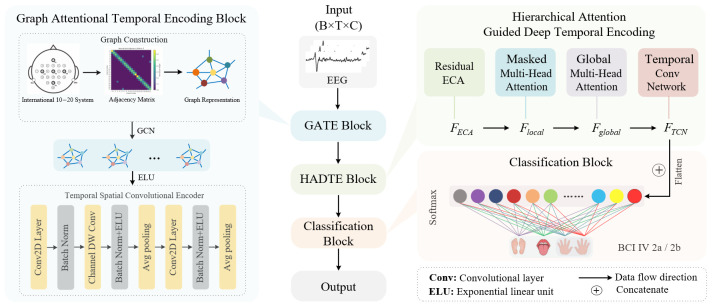
Overall architecture of GAH-TNet.

To address the above challenges, this paper proposes a deep neural network model for MI-BCI decoding—GAH-TNet. The overall architecture is shown in Figure 1. First, the model directly takes raw EEG signals as input and employs the GATE module to achieve collaborative modeling of initial spatiotemporal features. GATE is based on a graph neural network and incorporates a spatial connection graph constructed from the physical electrode layout to extract spatial structural features between channels using Chebyshev graph convolution. Through a structured spatio-temporal convolution module, it further models local temporal dynamics and cross-channel dependencies, yielding intermediate representations rich in contextual semantics. Subsequently, the HADTE module focuses on deep temporal modeling, implementing a hierarchical attention-guided mechanism to uncover multi-scale deep temporal dependencies. This module uses residual-ECA to enhance information interaction across channels. A local masked multi-head attention mechanism captures short-term dependencies, while a global multi-head attention mechanism models context across the entire temporal range. Subsequently, a temporal convolutional network is used for deep dynamic modeling, further expanding the temporal receptive field and enhancing feature stability. Finally, the high-order feature representations are fed into a classifier module composed of a fully connected layer and a softmax layer to generate the final category predictions. The main contributions of this paper are summarized as follows:

1. A novel deep neural network model, GAH-TNet, is proposed for MI decoding tasks. It integrates graph attention–based spatial structure and multi-level temporal feature encoding to achieve collaborative modeling of the spatial topology and dynamic temporal evolution of EEG signals.
2. The GATE module is designed to capture spatial dependencies and local temporal dynamic across EEG channels, enabling collaborative encoding of spatiotemporal features and enhancing both the structural integrity and discriminative power of feature representations.
3. We develop the HADTE module, which employs hierarchical attention guidance for cross-scale deep temporal modeling, significantly improving the ability of the model to perceive multi-stage deep temporal patterns during motor imagery.
4. We perform systematic experimental verification on the public BCI IV 2a and 2b datasets, where GAH-TNet consistently outperforms existing state-of-the-art models, demonstrating strong stability, generalization, and achieving SOTA performance.

## 2. Materials and Methods

### 2.1. Dataset

#### 2.1.1. BCI Competition IV Dataset 2a

**Figure 2:**
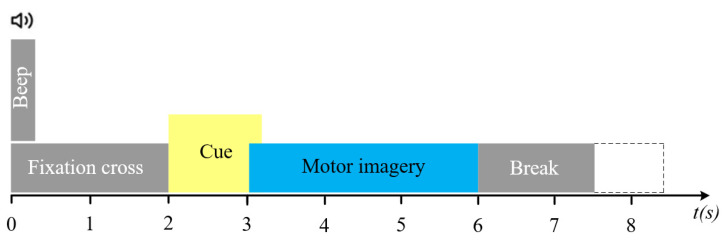
Movement imagination paradigm of BCI IV 2a.

The BCI IV 2a [36] dataset is a publicly available EEG motor imagery classification dataset released by Graz University of Technology in Austria in 2008. It is widely used for evaluating model performance in brain–computer interface research, with its experimental paradigm shown in Figure 2. The dataset includes EEG recordings from 9 healthy participants, each of whom completed four types of motor imagery tasks: left hand, right hand, both feet, and tongue, corresponding to four classification labels. EEG signals were recorded using 22 Ag/AgCl scalp electrodes conforming to the international 10–20 system, with a sampling rate of 250 Hz. Each subject underwent two rounds of data collection on different dates, designated as training and testing sessions, respectively. Each session comprised 288 trials (72 per category), with each trial lasting 4 s during the motor imagery task. The collected signals were processed through a bandpass filter with a bandwidth of 0.5–100 Hz. Due to the limited number of training samples and the presence of significant noise and artifacts in the signals, the classification task for this dataset presents a certain level of challenge. In this study, the data from the training session were used for model training, while the data from the testing session were used to evaluate model performance.

#### 2.1.2. BCI Competition IV Dataset 2b

The BCI IV 2b [37] dataset was also provided by Graz University of Technology and focuses on a binary classification motor imagery task, collected from nine healthy subjects. Each subject was required to complete a motor imagery task for either the left or right hand. EEG signals were recorded using three bipolar electrodes at C3, Cz, and C4, with a sampling rate of 250 Hz. The signals were processed through a bandpass filter with a bandwidth of 0.5–100 Hz. Each participant took part in five experimental sessions, with the first two sessions being feedback-free, each containing 120 trials; the last three sessions were feedback-based experiments, each containing 160 trials. In this study, we used the data from the first three sessions as the training set and the last two sessions as the test set to validate the model’s generalization ability across different experimental conditions and sessions.

### 2.2. Data Pre-Processing

The raw data in this study are represented in the form of C×T, where C denotes the number of electrode channels and T denotes the corresponding time sampling points. To avoid introducing prior information unrelated to the classification task, no additional filtering or feature engineering operations were performed during the preprocessing stage. Instead, the data from each channel were standardized to enhance training stability and eliminate scale effects. The standardization method employed Z-score normalization, with the calculation formula as follows:

(1) $$z=\frac{x-\mu }{\sqrt{\sigma^{2}}}$$

where x represents the original EEG signal, μ and $\sigma^{2}$ denote the mean and variance of each channel, respectively, and z is the normalized output value. In terms of time window selection, considering that EEG responses activate rapidly after the cue appears and have a short duration, the experiment extracted a complete 4.5-second data segment from 0.5 s before the cue appeared until the end of the trial for analysis. The sampling rate was 250 Hz, so each trial segment contained 250×4.5=1125 time points, resulting in an input data dimension of C×1125.

### 2.3. Model Architecture

This study proposes a novel deep neural network architecture for EEG motor imagery decoding, named GAH-TNet, which aims to simultaneously capture the spatial topological structure and deep temporal dependency characteristics of EEG signals. The overall architecture is illustrated in Figure 1. GAH-TNet comprises three core modules: the graph-attentional temporal encoding block (GATE), the hierarchical attention-guided deep temporal encoder (HADTE), and the classifier module. Its design follows a “spatial–temporal collaborative modeling, hierarchical attention guidance, and deep temporal modeling” approach, significantly enhancing the classification performance of motor imagery EEG data. Detailed descriptions of each module are provided below.

#### 2.3.1. Graph-Attentional Temporal Encoding Block

To effectively model the spatial dependencies between channels in EEG signals and the dynamic feature changes in time series, this study proposes GATE-Block as the initial feature extraction module of the model. This module takes the raw EEG signal $X\in R^{T\times C}$ as input. Through four stages—constructing a spatial connectivity graph, graph convolution feature extraction, channel attention enhancement, and temporal modeling—the module ultimately produces a high-level feature representation $F_{GATE}\in R^{T^{\prime }\times C}$ that integrates structural and dynamic information. The details of each component of the module are described below.

(1) Graph Construction and Normalization

First, based on the physical connection relationships of EEG electrodes in spatial layout, a two-dimensional adjacency graph G=(V,E) is constructed between channels. Each channel is a graph node, and edges between nodes are connected according to electrode adjacency relationships and their physical locations, thereby ensuring that the graph structure reasonably reflects the spatial proximity and physiological characteristics of scalp electrodes. The adjacency relationship is represented by an adjacency matrix $A\in R^{C\times C}$.

(2) $$A_{ij}=\left\{\begin{array}{c} 1,\;if\;i=j\;or\;\left(i,j\right)\;\epsilon \;E\\ 0,\;otherwise \end{array}\right.$$

To enhance numerical stability in the graph convolutional network and avoid the unbalanced impact of node degrees on information propagation, the adjacency matrix is subjected to symmetric normalization, calculated as follows:

(3) $$\hat{A}={\tilde{D}}^{-\frac{1}{2}}\left(A+I\right){\tilde{D}}^{-\frac{1}{2}}$$

where $\tilde{D}$ is the degree matrix of A+I, and I is the identity matrix. The normalized $\hat{A}$ provides a standardized topological structure foundation for subsequent graph convolution modules, enabling the model to extract more effective spatial dependency features under the constraints of the physical electrode topological structure.

(2) Chebyshev Graph Convolution

The original input signal $X\in R^{T\times C}$ is regarded as a node feature sequence of length T, and kth-order Chebyshev graph convolution is introduced to extract structural features. In this study, k is set to 3. For each time step t, the graph convolution is represented as follows:

(4) $$Z_{t}=\sum_{k=0}^{K-1}T_{k}(\hat{A})X_{t}W_{k}$$

where $X_{t}\in R^{C}$ is the feature of all channels at time t, $W_{k}$ is the learnable weight of the kth-order Chebyshev convolution, and $T_{k}(\cdot )$ is the *k*th-order Chebyshev polynomial. We can stack the convolution results of all time steps to obtain the graph feature sequence shown below:

(5) $$Z=[Z_{1};Z_{2};\ldots ;Z_{T}]\in R^{T\times d_{g}}$$

where $d_{g}$ denotes the channel feature dimension after graph convolution.

(3) Channel Attention

To further enhance the response to important channels in the graph convolution results, this paper introduces a channel attention module based on the squeeze-and-excitation (SE) mechanism [38] to dynamically weigh Z. This module first extracts channel statistical information through global pooling:

(6) $$s=AvgPool(Z)\in R^{d_{g}}$$

Then, it generates attention weights through two layers of fully connected networks and nonlinear activation functions:

(7) $$w_{c}=\sigma (W_{2}\cdot ReLU(W_{1}\cdot s))$$

Finally, the graph convolution results are weighted channel-wise to obtain the enhanced feature representation:

(8) $$Z^{\prime }=Z⊙w_{c}$$

where ⊙ denotes channel-wise multiplication.

(4) Temporal–Spatial Convolutional Encoder

We introduce a structured temporal–spatial convolutional encoding module on the output $Z^{\prime }$ enhanced by graph attention, further modeling the dynamic changes in EEG signals at different temporal scales and enhancing the model’s ability to integrate cross-channel spatial dependencies. Inspired by the EEGNet network architecture, this module consists of three consecutively stacked convolutional, normalization, activation, and pooling structures, forming a multi-layer convolutional block aimed at capturing local, deep, and global temporal dependency patterns.

First, temporal convolution uses a two-dimensional convolution kernel $\left(k_{t},1\right)$ unfolded along the time axis to independently model the local temporal dynamic features of each channel. Specifically, it is expressed as follows:

(9) $$H_{1}=BN\left(Conv2D_{\left(k_{t},1\right)}(Z^{\prime })\right)$$

where the convolution kernel size is set to $k_{t}=64$, and the number of output channels is $F_{1}=16$. Then, the channel depthwise spatial convolution is used to model cross-channel spatial dependencies. The module employs a depthwise separable convolution kernel (C,1) to compress and aggregate the coupling relationships between channels in the channel dimension. The computational expression is as follows:

(10) $$H_{2}=ELU(BN(DepthwiseConv2D_{\left(C,1\right)}(H_{1})))$$

Each input channel is mapped to two feature maps, with a total of $F_{2}=32$ output channels. A $\left(8,1\right)$ average pooling layer is used to compress the temporal dimension. To further expand the model’s receptive field, a $\left(16,1\right)$ 2D convolution is used to model long-term dependencies and global contextual dynamics. Its output is defined as follows:

(11) $$H_{3}=Dropout(AvgPool_{\left(p,1\right)}(ELU(BN(Conv2D_{\left(16,1\right)}(H_{2}))))$$

where the pooling window size is set to P=7. Finally, the high-order spatio-temporal feature representation sequence is obtained as follows:

(12) $$F_{GATE}=H_{3}\in R^{T^{\prime }\times F_{2}}$$

where $T^{\prime }$ denotes the sequence length after two temporal pooling operations.

#### 2.3.2. Hierarchical Attention-Guided Deep Temporal Feature Encoding Block

To further strengthen the model’s ability to model the temporal dynamics of EEG signals and improve the discriminative power of feature representations, this paper designs the HADTE-Block. The module receives the intermediate feature representation $F_{GATE}$ from the GATE output and, based on this, extracts temporal dependencies at different scales, ultimately forming high-level abstract features with global perception capabilities and local discriminative power. The specific details are as follows.

(1) Residual-ECA

**Figure 3:**
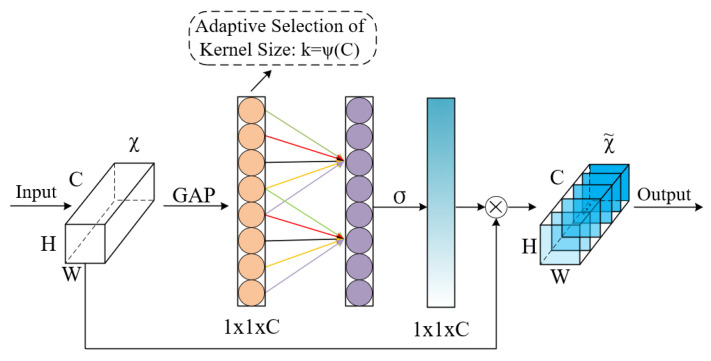
ECA architecture, where the dimensions of the feature map are represented by parameters C, H, and W. GAP stands for global average pooling, and ⊗ represents element-wise product.

First, the efficient channel attention (ECA) mechanism is introduced to enhance the input features $F_{GATE}$. ECA [39] replaces explicit fully connected operations with local one-dimensional convolutions, using an adaptive weighting strategy based solely on cross-channel information to strengthen the response to important feature channels. Its structural design is shown in Figure 3. We adopt a residual connection form to preserve the original feature path [40], with the calculation formula as follows:

(13) $$F_{eca}=F_{GATE}+ECA(F_{GATE})$$

Residual-ECA significantly enhances the model’s ability to perceive implicit dependencies between channels while maintaining computational efficiency and enhancing context sensitivity.

(2) Local Masked Multi-Head Attention

To capture short-term dependency patterns in EEG signals, we employ a multi-head self-attention mechanism based on a local-aware mask, guiding each time step to focus solely on its local neighborhood within the window range. Specifically, for any two time steps i and j, the attention mask $M_{ij}$ is defined as follows:

(14) $$M_{ij}=\left\{\begin{array}{c} 0,\;if\;|i-j|\leq w\cdot d\\ -\infty ,\;otherwise \end{array}\right.$$

where w is the window size, set to 16, and d is the dilation rate, set to 1. This strategy is equivalent to introducing a positional selectivity penalty term into the attention weights, allowing only tokens within the window range to participate in the attention calculation. The output of the local attention is as follows:

(15) $$F_{local}=MHA\left(F_{eca},F_{eca},F_{eca},mask=M\right)+F_{eca}$$

Local masked attention modeling has good local dynamic modeling capabilities while retaining the global learnability of the attention mechanism.

(3) Global Multi-Head Attention

**Figure 4:**
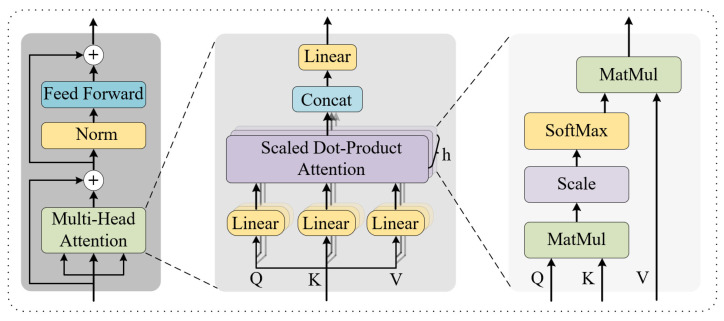
Architecture diagram of the multi-head self-attention mechanism.

Based on local modeling, we further utilize the multi-head self-attention mechanism to achieve long-term dependency modeling across the entire sequence, as shown in Figure 4. To stabilize the feature distribution, the input is first processed through layer normalization:

(16) $$F_{global}=MHA\left({LN(F}_{local}),{LN(F}_{local}),{LN(F}_{local})\right)+F_{local}$$

This mechanism allows free modeling of attention weights between any two time steps, thereby capturing long-range semantic dependencies and enhancing the ability to focus on task-critical events [41]. The serial fusion of local and global attention achieves cross-scale temporal perception. Experimental results show that the model’s feature learning ability is optimal when using two-headed attention with each attention head dimension set to 8.

(4) Deep temporal modeling

**Figure 5:**
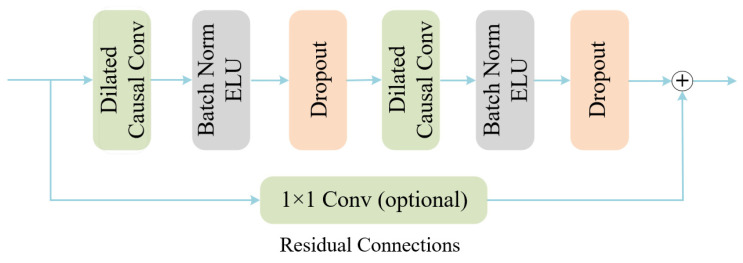
Temporal convolutional networks.

To further enhance the adaptability to non-stationary temporal changes in EEG, we introduce a temporal convolutional network (TCN) on top of the global attention features for deep temporal modeling, as shown in Figure 5. TCN constructs a large receptive field by introducing dilated convolution and residual blocks, while maintaining causality and sequence alignment:

(17) $$F_{TCN}=TCN(F_{global})$$

#### 2.3.3. Classifier Module

At the final stage of the model, a classifier module is employed to determine the task category. This module consists of a fully connected neural network layer that maps the high-level embedding vector $F_{TCN}$, generated by the preceding feature extraction module, onto the category space. The output of this layer is then passed through a softmax activation function to produce the predicted probability distribution across categories. The mathematical form of the Softmax function is as follows:

(18) $$P(y_{i})=\frac{e^{z_{i}}}{\sum_{j=1}^{M}e^{z_{j}}}$$

where $P(y_{i})$ represents the probability that the sample belongs to the i-th category, $z_{i}$ is the i-th component of the output from the fully connected layer, and M is the total number of categories. This function normalizes the original output into a probability distribution, making it easier to represent the model’s confidence in predicting each category.

To facilitate the learning of effective decision boundaries during training, the cross-entropy loss function is adopted as the optimization objective. This function quantifies the discrepancy between the predicted class probabilities and the ground-truth labels, and its formulation is given as follows:

(19) $$L=-\frac{1}{N_{b}}\sum_{n=1}^{N_{b}}\sum_{m=1}^{M}y_{n,m}log({\hat{y}}_{n,m})$$

where $y_{n,m}$ and ${\hat{y}}_{n,m}$ represent the true label and predicted probability of the nth sample in the mth class, respectively, and $N_{b}$ is the number of samples in each batch. This loss function effectively improves the model’s classification accuracy by taking the negative logarithm of the predicted probability and matching it with the true label. To achieve a stable and efficient training process, this study uses the Adam optimization algorithm for adaptive updates of model parameters.

### 2.4. Evaluation Metrics

To comprehensively evaluate the model’s decoding capability in the EEG signal classification task, this paper uses classification accuracy and the Kappa coefficient as the primary performance metrics. These two metrics are widely used evaluation standards in EEG-related research, serving to measure the model’s overall prediction accuracy and category consistency level, respectively.

Classification accuracy (*ACC*) reflects the proportion of correctly predicted samples in the entire dataset, defined as follows:

(20) $$ACC=\frac{TP\;+\;TN}{TP\;+\;TN\;+\;FP\;+\;FN}$$

where *TP* denotes the number of samples predicted as positive and actually positive (true positives), *TN* denotes the number of samples predicted as negative and actually negative (true negatives) and *FP* and *FN* represent false positives and false negatives, respectively. The accuracy value ranges from 0 to 1, with higher values indicating stronger discriminative ability of the model.

The Kappa coefficient is used to measure the consistency between the model’s predicted results and the actual labels, eliminating the influence of random consistency. Its formula is shown below:

(21) $$\kappa =\frac{p_{o}-p_{e}}{1-p_{e}}$$

where $p_{o}$ represents the observed consistency probability, and $p_{e}$ represents the consistency probability under the assumption of random guessing. The Kappa coefficient ranges from −1 to 1, with values closer to 1 indicating higher consistency between the model’s predictions and the actual situation.

Additionally, to further test whether the performance differences between different methods are statistically significant, this paper uses the Wilcoxon signed-rank test for nonparametric significance analysis of paired samples. This method does not require the data distribution to be normal and is suitable for small samples or non-Gaussian distributions, making it robust in EEG data analysis. When *p* > 0.05, the difference is not significant; *p* < 0.05 (denoted as *) indicates statistical significance; and *p* < 0.01 (denoted as **) indicates a highly significant difference. This test provides rigorous statistical support for validating the effectiveness of the model improvement strategy.

## 3. Experiments and Results

### 3.1. Experiment Details

All experiments were conducted on a single NVIDIA GeForce RTX 2080Ti GPU(NVIDIA Corporation, Santa Clara, CA, USA), with the model implemented using the TensorFlow framework. During model training, the Adam optimizer was used with an initial learning rate of 0.001, a batch size of 64, and a total of 1000 training iterations. To prevent overfitting, an early stopping strategy was introduced with a patience parameter set to 300. In the experiment, all hyperparameters were manually adjusted through multiple rounds of trials to achieve better classification performance and model generalization ability.

**Figure 6:**
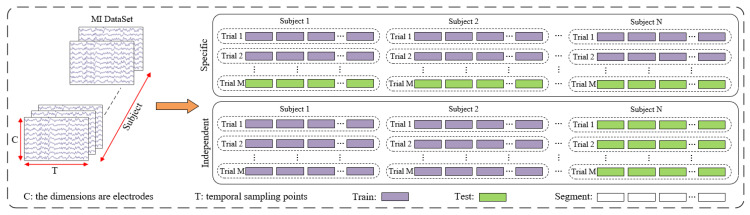
Framework diagram of subject-specific and subject-independent. Each dataset contains N subjects, and each subject has M trials.

GAH-TNet was evaluated using both subject-dependent and subject-independent experiments, as illustrated in Figure 6. For subject-dependent experiments, the first session of BCI IV 2a was used for training, and the second session was used for testing. The first three sessions of BCI IV 2b were used for training, and the last two sessions were used for testing. This experiment divides the training and testing sets based on subjects and trials, effectively avoiding information leakage. For subject-independent experiments, the leave-one-subject-out cross-validation (LOSO-CV) evaluation method was adopted, which effectively verifies individual differences among subjects and the model’s generalization ability. One subject was selected from the dataset as the testing set, and the data from the remaining subjects were used as the training set. This process was repeated until all subjects’ data had been tested.

### 3.2. Results

#### 3.2.1. Experimental Results of MI-EEG Decoding

To evaluate the performance of the proposed GAH-TNet model in motor imagery EEG decoding tasks, we conducted evaluations on the publicly available BCI IV 2a and 2b datasets. Table 1 presents the classification accuracy and Kappa values under both subject-dependent and subject-independent settings.

On the BCI IV 2a dataset, GAH-TNet achieved an average classification accuracy of 86.84% and a Kappa value of 0.82 in the subject-dependent setting. The best-performing subject was A03, with an accuracy of 97.22% and a Kappa of 0.96. In the LOSO setting, although the overall accuracy decreased, the average value still reached 69.39%, with a Kappa value of 0.59, demonstrating robust generalization ability across subjects. Similarly, on the BCI IV 2b dataset, GAH-TNet still exhibited the best decoding capability. Under subject-related conditions, the model achieved an average accuracy rate of 89.15% and a Kappa value of 0.78, with accuracy rates exceeding 94% for subjects A04, A05, and A08. In the LOSO setting, the average accuracy rate was 81.00%, with a Kappa value of 0.62, further confirming the cross-subject robustness of GAH-TNet.

The experimental results demonstrate that the two core modules, GATE and HADTE, effectively collaborate in feature modeling. The GATE module employs graph attention convolutions to capture the spatial topological structure and primary temporal features across EEG channels, while the HADTE module further enhances representation of non-stationary EEG signals through hierarchical attention-guided deep temporal feature modeling. GAH-TNet achieves superior classification performance on two mainstream MI-EEG datasets, validating the rationale and necessity of its architectural design.

**Figure 7:**
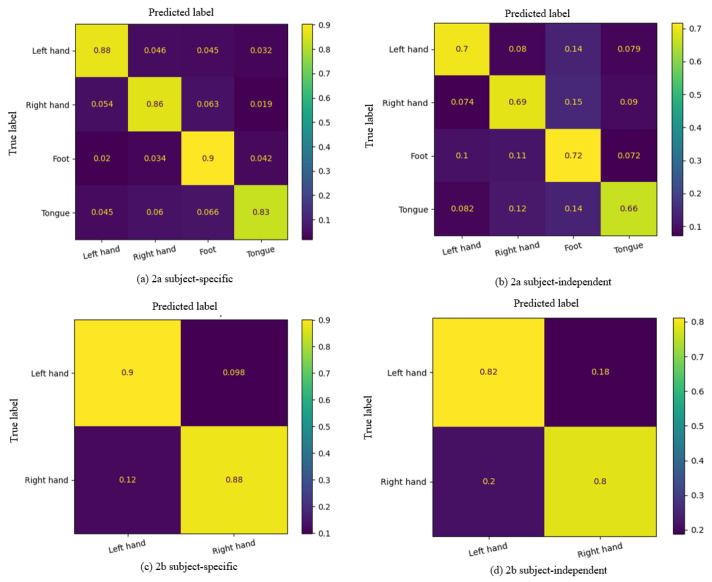
Average confusion matrix of GAH-TNet: (**a**) BCI IV 2a subject-related, (**b**) BCI IV 2a subject-independent, (**c**) BCI IV 2b subject-related and (**d**) BCI IV 2b subject-independent matrix.

The average confusion matrix of GAH-TNet on the two datasets is shown in Figure 7. In the BCI IV 2a dataset, GAH-TNet demonstrated high recognition ability for all four task categories under the subject-related setting (Figure 7a). The recognition accuracy for the “Foot” category is the highest, reaching 90%; confusion rates are generally low, indicating that the model can effectively distinguish between complex multi-category motor imagery tasks in subject-related scenarios. In the subject-independent setting (Figure 7b), although the overall accuracy decreases due to challenges posed by individual differences, GAH-TNet still maintains stable classification performance. The “Tongue” category had the lowest recognition rate (66%) and showed some confusion with ‘Foot’ and “Right hand,” possibly due to the similarity of EEG features across different subjects for these categories. Overall, the confusion matrix distribution in this setting remained relatively balanced, validating the good cross-subject generalization of the model.

On the BCI IV 2b dataset, which involves only the “Left hand” and “Right hand” categories, GAH-TNet demonstrates higher classification performance. Under the subject-related setting (Figure 7c), the classification accuracy rates for “Left hand” and “Right hand” reach 90% and 88%, respectively, with an extremely low misclassification rate, indicating effective distinguish between the two hand movement imagery categories. In the subject-independent setting (Figure 7d), although the accuracy rate decreased slightly, the overall performance remained excellent. Compared to the 2a dataset, GAH-TNet still demonstrated strong robustness and interference resistance even with fewer channels.

**Figure 8:**
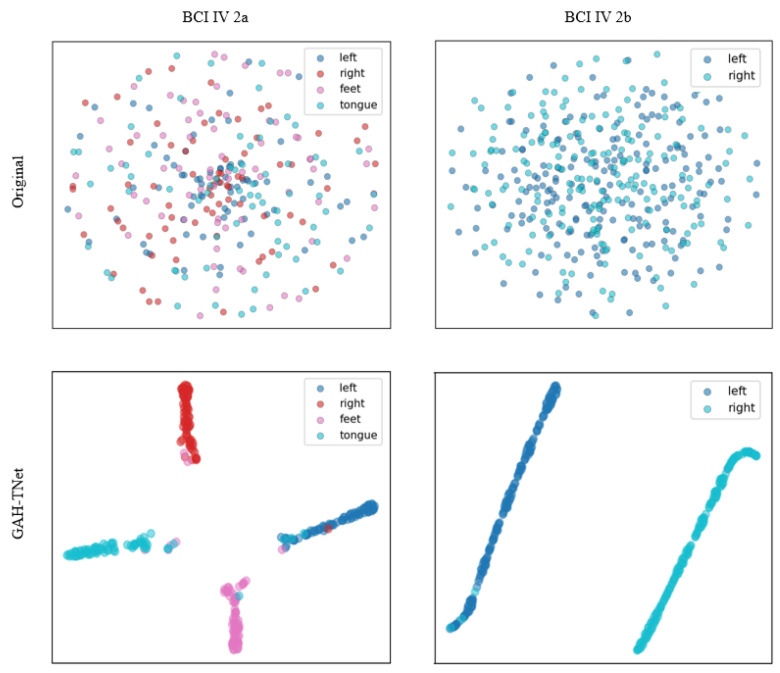
t-SNE visualizations of different datasets.

Figure 8 shows the t-SNE [42] dimensionality reduction visualization of the original input features and the features extracted by the GAH-TNet model in a two-dimensional space for two datasets. It is evident that the GAH-TNet model demonstrates significant discriminative capability for EEG features. For the BCI IV 2a dataset, the original features exhibit highly intermingled distributions of samples across categories, with blurred category boundaries. After encoding by GAH-TNet, the four categories of motor imagery samples form clearly separated clusters in the feature space, demonstrating the model’s excellent discriminative and embedding capabilities. For the BCI IV 2b dataset, the “left” and “right” categories in the original features are highly overlapping, while the features extracted by GAH-TNet exhibit clear linear separability, significantly enhancing the separability between different categories. Overall, the model can extract deep feature representations with good structural integrity and discriminative power from complex EEG signals, further validating its superior performance in motor imagery decoding tasks.

#### 3.2.2. Ablation Experiment

To evaluate the contribution of each key module in GAH-TNet, we conducted systematic ablation experiment. The classification accuracy and Kappa values after removing different units are shown in Table 2. In the BCI IV 2a dataset, the GAH-TNet model achieved the highest accuracy of 86.84% and Kappa value of 0.82. Removing Channel Attention resulted in a slight decrease in performance (85.22%, 0.79), indicating that this module effectively emphasizes spatial features. Excluding the Temporal–Spatial Convolutional Encoder caused the accuracy rate to drop to 79.68% and the Kappa value to 0.72, indicating that it plays a critical role in initial feature modeling.

Additionally, removing Residual-ECA and Local Masked Multi-Head Attention modules led to accuracy reductions to 85.53% and 83.65%, respectively, indicating their synergistic contribution to extracting fine-grained temporal features. Excluding Global Multi-Head Attention also caused performance degradation (84.47%), further validating the complementary nature of local and global modeling. When both local and global attention mechanisms are removed simultaneously, model performance further decreases to 82.64%, highlighting the importance of hierarchical attention fusion design in temporal modeling. Notably, removing the entire HADTE module results in a significant accuracy drop to 80.29% with a Kappa of 0.73, indicating the module’s irreplaceable role in capturing multi-scale temporal dependencies. In the BCI IV 2b dataset, the ablation trends are consistent with those in 2a, and further details are omitted here.

The experimental results indicate that the key module of GAH-TNet are essential for its performance. In particular, the HADTE module and the spatio-temporal convolutional encoder substantially enhance the temporal modeling capabilities of the model, while the attention mechanism further enhances the flexibility and expressiveness of feature selection. These design units collectively support GAH-TNet’s superior performance in MI-EEG decoding tasks.

#### 3.2.3. Comparison of GAH-TNet with Other Models

To comprehensively assess the reliability and advancement of the proposed GAH-TNet in EEG signal classification, several representative comparison models were selected for systematic evaluation. These include classical convolutional neural network models (such as Shallow ConvNet and EEGNet), deep models with temporal modeling capabilities (such as EEG-TCNet and TCNet Fusion), and advanced structures incorporating attention mechanisms introduced in recent years (such as Conformer and EEG-TransNet). Collectively, these models cover a spectrum of modeling strategies—from shallow to deep, convolutional to temporal, and local to global—demonstrating both representativeness and broad applicability.

As shown in Table 3 and Table 4, the proposed GAH-TNet model achieves the highest classification accuracy and Kappa values on both the BCI IV 2a and BCI IV 2b datasets, outperforming other comparison methods in both subject-specific and subject-independent settings.

For the 2a dataset, under the subject-specific setting, GAH-TNet achieved an accuracy/Kappa of 86.84%/0.82, representing an improvement of 3.8 percentage points over the second-best EEG-TransNet (83.03%/0.78). Although the improvement is relatively modest, given the small differences between individual subject samples and the ease of model fitting under the subject-specific setting, overall performance is already close to saturation. GAH-TNet still achieves a robust performance, highlighting the effectiveness of its structural design; Under the subject-independent setting, GAH-TNet’s advantage is even more pronounced, achieving 69.39%/0.59, nearly 5 percentage points higher than EEG-TransNet (64.53%/0.50). Cross-subject scenarios place higher demands on the model’s spatio-temporal feature modeling capabilities, and this gap indicates that GAH-TNet has a clear advantage in terms of generalization ability.

For the 2b dataset, in the subject-specific setting, GAH-TNet achieved an accuracy/Kappa of 89.15%/0.78, outperforming EEG-TransNet (88.10%/0.76) and TCNet Fusion (87.61%/0.74). Although the gap with the best baseline method is not significant, it is important to note that EEG data exhibits high non-stationarity. The fact that GAH-TNet consistently maintains a stable lead under such conditions indicates that it captures task-critical patterns with greater precision; In the subject-independent setting, GAH-TNet performed most notably, achieving accuracy and Kappa values of 81.00%/0.62, respectively, which are significantly higher than those of EEG-TransNet (79.68%/0.58) and TCNet Fusion (77.53%/0.53). This further demonstrates that GAH-TNet has stronger capabilities in modeling complex spatio-temporal dependencies across subjects, exhibiting superior robustness.

From a model structure perspective, GAH-TNet has the following key advantages over other comparison methods:

• Advantages over traditional CNN models in spatial dependency modeling: Shallow ConvNet and EEGNet primarily rely on convolution operations to extract local features, making it difficult to model non-local spatial dependencies between channels. In contrast, GAH-TNet introduces a GCN module via GATE, effectively leveraging the topological structure of EEG electrodes to model cross-channel spatial relationships, enabling the model to uncover latent connections from structured graph spaces. In cross-subject experiments, GAH-TNet outperformed EEGNet by 12.6 percentage points on the 2a dataset, validating the critical role of its spatial modeling capabilities in generalization performance.
• Advantages over Transformer models in hierarchical temporal modeling mechanisms: EEG-Conformer and EEG-TransNet often emphasize global attention or single-time-domain modeling, which may overlook critical local time segments. GAH-TNet introduces local-aware masked attention in the HADTE module, highlighting local dynamic patterns by restricting the attention window while simultaneously achieving joint modeling of global semantics. In the subject-specific setting of the 2b dataset, GAH-TNet achieves a 1.05 percentage point improvement over EEG-TransNet at near-saturated performance levels, indicating that its hierarchical temporal modeling mechanism can capture more discriminative features in critical time segments.
• Advantages over fusion models in spatiotemporal synergy: TCNet Fusion and EEG-TCNet address feature diversity to some extent but still lack effective modeling of spatio-temporal dependencies. Under the subject-independent setting of the 2a dataset, GAH-TNet achieved an accuracy of 69.39%, representing a 6.1 percentage point improvement over TCNet Fusion (63.30%), and on the 2b dataset, it maintained a 3.5 percentage point advantage. These results indicate that GAH-TNet not only leverages the long-range temporal modeling capability of TCN but also enhances feature representation through the synergistic effects of graph attention and spatiotemporal convolutional encoding, thereby improving the spatiotemporal resolution and decoding performance of EEG signals.

In summary, the superior performance of GAH-TNet across multiple comparative experiments results from its effective deep fusion modeling across spatial, channel, and temporal dimensions, which provides the model with enhanced expressive power and robust cross-subject generalization in complex EEG recognition tasks. These results further highlight the significant potential of graph attention–guided hierarchical temporal feature modeling in EEG decoding.

**Figure 9:**
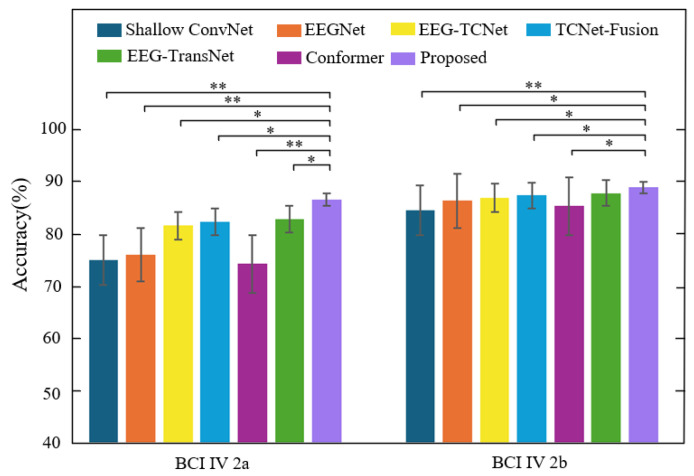
Performance comparison between the proposed method and other methods on two datasets (subject-related), *: *p* < 0.05, **: *p* < 0.01.

Figure 9 shows the subject-related experimental results of each model on the two datasets and evaluates the performance differences through significance testing. As shown, the proposed method exhibits significant differences from multiple baseline models on both datasets. Specifically, on the BCI IV 2a dataset, the performance differences between the proposed method and methods such as Shallow ConvNet, EEGNet, Conformer, and EEG-TransNet reach a highly significant level (**), indicating that it possesses statistically reliable advantages in subject-specific classification tasks. On the BCI IV 2b dataset, the method also exhibits significant and highly significant differences compared to mainstream comparison models such as EEGNet, EEG-TCNet, and Conformer, further validating its stability and robustness in multi-subject EEG decoding scenarios.

## 4. Conclusions

This paper presents GAH-TNet, a novel graph attention-based hierarchical temporal network for decoding motor imagery electroencephalogram signals. The model integrates a graph attention mechanism to capture spatial dependencies across EEG channels and employs a hierarchical attention mechanism to guide the temporal encoder, thereby extracting multi-scale temporal features that capture both short-term and long-term dependencies. Extensive experimental results on the BCI IV 2a and 2b datasets demonstrate that GAH-TNet consistently outperforms existing state-of-the-art models in terms of classification accuracy and generalization ability. Ablation experiments further validate the complementary roles of the graph attention module and the temporal encoding module in performance improvement. By effectively modeling both the spatial topological structure and temporal dynamic features of EEG signals, GAH-TNet provides a stable, interpretable, and well-generalized decoding framework with strong potential for practical applications in brain–computer interface systems. Future work will focus on evaluating the robustness and adaptability of GAH-TNet in cross-task transfer scenarios, facilitating its application to real-world complex tasks and personalized BCI systems.

## Figures and Tables

**Table 1:** Classification accuracy and Kappa values of GAH-TNet on the BCI IV 2a and BCI IV 2b datasets.

Subject	BCI IV 2a				BCI IV 2b			
	GAH-TNet		GAH-TNet (LOSO)		GAH-TNet		GAH-TNet (LOSO)	
	%	*k*	%	*k*	%	*k*	%	*k*
A01	90.97	0.88	76.74	0.69	80.94	0.62	80.28	0.60
A02	76.39	0.69	53.30	0.38	73.57	0.48	74.71	0.49
A03	97.22	0.96	86.11	0.81	88.44	0.76	67.64	0.35
A04	84.38	0.79	63.02	0.50	97.81	0.95	86.76	0.74
A05	81.25	0.75	59.38	0.46	94.69	0.89	87.97	0.76
A06	75.69	0.67	53.82	0.38	90.31	0.81	83.47	0.66
A07	96.18	0.95	72.05	0.63	92.81	0.85	85.83	0.71
A08	88.19	0.84	82.99	0.77	95.31	0.91	82.50	0.65
A09	91.32	0.88	77.08	0.69	88.44	0.76	79.86	0.60
Mean	86.84	0.82	69.39	0.59	89.15	0.78	81.00	0.62

**Table 2:** Ablation experiment results of GAH-TNet on the BCI IV 2a and BCI IV 2b datasets, where “w/o” indicates the absence of the corresponding module.

Dataset	Method	Accuracy (%)	Kappa
BCI IV 2a	w/o Channel Attention	85.22	0.79
	w/o Temporal-Spatial Convolutional Encoder	79.68	0.72
	w/o Residual-ECA	85.53	0.80
	w/o Local Masked Multi-Head Attention	83.65	0.77
	w/o Global Multi-Head Attention	84.47	0.79
	w/o Local Masked Multi-Head Attention and Global Multi-Head Attention	82.64	0.76
	w/o HADTE	80.29	0.73
	GAH-TNet	86.84	0.82
BCI IV 2b	w/o Channel Attention	87.85	0.76
	w/o Temporal-Spatial Convolutional Encoder	81.04	0.63
	w/o Residual-ECA	87.44	0.74
	w/o Local Masked Multi-Head Attention	86.31	0.72
	w/o Global Multi-Head Attention	87.54	0.75
	w/o Local Masked Multi-Head Attention and Global Multi-Head Attention	84.66	0.70
	w/o HADTE	83.25	0.68
	GAH-TNet	89.15	0.78

**Table 3:** Classification accuracy, Kappa value, and *p*-value of the method described in this paper and each comparison method on the BCI IV 2a dataset. Bold denotes the best numerical values.

Dataset	Method	Accuracy (%)	Kappa
Subject-specific	Shallow ConvNet	75.25	0.66
	EEGNet	76.25	0.68
	Conformer	74.51	0.65
	EEG-TCNet	81.84	0.75
	TCNet Fusion	82.53	0.76
	EEG-TransNet	83.03	0.78
	GAH-TNet	**86.84**	**0.82**
Subject-independent	Shallow ConvNet	55.03	0.40
	EEGNet	56.80	0.42
	Conformer	55.25	0.40
	EEG-TCNet	62.26	0.49
	TCNet Fusion	63.30	0.49
	EEG-TransNet	64.53	0.50
	GAH-TNet	**69.39**	**0.59**

**Table 4:** Classification accuracy, Kappa value, and *p*-value of the method described in this paper and each comparison method on the BCI IV 2b dataset. Bold denotes the best numerical values.

Dataset	Method	Accuracy (%)	Kappa
Subject-specific	Shallow ConvNet	84.82	0.70
	EEGNet	86.63	0.71
	Conformer	85.63	0.70
	EEG-TCNet	87.20	0.74
	TCNet Fusion	87.61	0.74
	EEG-TransNet	88.10	0.76
	GAH-TNet	**89.15**	**0.78**
Subject-independent	Shallow ConvNet	75.48	0.51
	EEGNet	76.33	0.53
	Conformer	73.26	0.46
	EEG-TCNet	77.08	0.52
	TCNet Fusion	77.53	0.53
	EEG-TransNet	79.68	0.58
	GAH-TNet	**81.00**	**0.62**

## Data Availability

The two datasets used in this study, namely BCI Competition IV 2a and 2b, are publicly available and were used for experimental validation. These data can be found here: https://www.bbci.de/competition/download/competition_iv/BCICIV_2a_gdf.zip (accessed on 31 July 2025). https://www.bbci.de/competition/download/competition_iv/BCICIV_2b_gdf.zip (accessed on 31 July 2025).
